# Supplementary material for: Complex patterns of multimorbidity associated with severe COVID-19 and long COVID
Source: Commun Med (Lond). 2024 Jul 8;4:94. doi: 10.1038/s43856-024-00506-x (PMC11231221; doi:10.1038/s43856-024-00506-x)
Supplement: Supplementary file 2 — Description of Additional Supplementary Files [file 43856_2024_506_MOESM2_ESM.pdf]

Description of Additional Supplementary Files

File name- Supplementary Data 1

File description- Case numbers and definitions for survival and genetic analysis

File name- Supplementary Data 2

File description- Results from Coxproportional hazard analysis considering each disease as an exposure and time to each of four COVID-19 definition as an outcome. P-prop. hazard= p-value testing the proportional hazard assumption; NA = model did not converge

File name- Supplementary Data 3

File description- Results from sensitivity analysis considering competing risk models and additional adjustment of Cox-proportional hazard models.

File name- Supplementary Data 4

File description- Results from interaction terms in Cox-proportional hazard models.

File name- Supplementary Data 5a

File description- Disease-disease network based on significant partial correlations.

File name- Supplementary Data 5b

File description- Nodes characteristics of the disease-disease network

File name- Supplementary Data 6

File description- Results from genetic correlation analysis

File name- Supplementary Data 7

File description- Results from Mendelian randomization analysis

File name- Supplementary Data 8

File description- Results from diseasomewide colocalisation analysis at COVID-19 risk loci; R2.group = cluster of genetic variants tagging the regional sentinel for COVID-19; NEA = non-effect allele; EA = effect allele; EAF = effect allele frequency;
